# Short-term electrical stimulation promotes partial functional and morphological maturation of human-induced pluripotent stem cell-derived cardiomyocytes enabling cardiotoxicity risk mitigation at early-stage drug discovery for cardiac contractility modulation

**DOI:** 10.1093/toxsci/kfag018

**Published:** 2026-02-19

**Authors:** Hayato Miyoshi, Kaoru Morimura, Reiko Hara, Ritsuko Hori, Eriko Watanabe, Nobuyuki Mochizuki, Ayako Kamei, Rika Yamazaki, Katsuyuki Kazusa

**Affiliations:** Bioscience and Engineering Laboratories, Fujifilm Corporation, Ashigarakami, Kanagawa 258-8577, Japan; Consortium for Safety Assessment using Human iPS cells (CSAHi), Japan; Bioscience and Engineering Laboratories, Fujifilm Corporation, Ashigarakami, Kanagawa 258-8577, Japan; Consortium for Safety Assessment using Human iPS cells (CSAHi), Japan; Bio Business Promotion Department, Medical Division, NIKKISO Co., Ltd, Shibuya, Tokyo 150-6022, Japan; Bioscience and Engineering Laboratories, Fujifilm Corporation, Ashigarakami, Kanagawa 258-8577, Japan; Consortium for Safety Assessment using Human iPS cells (CSAHi), Japan; Analysis Technology Center, Fujifilm Corporation, Minamiashigara, Kanagawa 250-0193, Japan; Bioscience and Engineering Laboratories, Fujifilm Corporation, Ashigarakami, Kanagawa 258-8577, Japan; Consortium for Safety Assessment using Human iPS cells (CSAHi), Japan; Consortium for Safety Assessment using Human iPS cells (CSAHi), Japan; Toyama Research and Development Center, FUJIFILM Toyama Chemical Co. Ltd, Toyama, Toyama, 930-8508, Japan; Bioscience and Engineering Laboratories, Fujifilm Corporation, Ashigarakami, Kanagawa 258-8577, Japan; Consortium for Safety Assessment using Human iPS cells (CSAHi), Japan; Analytical Development, Moderna Enzymatics Co., Ltd, Fujisawa, Kanagawa, 251-0012, Japan; Consortium for Safety Assessment using Human iPS cells (CSAHi), Japan; Axion BioSytems, Inc, Atlanta, GA 30363, United States; Consortium for Safety Assessment using Human iPS cells (CSAHi), Japan; Applied Research and Non-Clinical Safety, Early Development and Translational Science, Astellas Pharma IncTsukuba, Ibaraki, 305-8585, Japan

**Keywords:** cardiovascular toxicity, cardiomyocytes, induced pluripotent stem cell, electric stimulation, myocardial contraction, multielectrode array recordings

## Abstract

In drug discovery, assessing cardiac contractile force is crucial because of its association with the development of cardiovascular events and heart failure. Human induced pluripotent stem cell-derived cardiomyocytes (hiPSC-CMs) provide a promising in vitro model for drug discovery, particularly for assessing proarrhythmic risk. However, the availability of robust in vitro models to evaluate cardiac contractility has been limited. Here, we demonstrate that subjecting hiPSC-CMs to electrical stimulation for 48 h using a multielectrode array system induces partial functional and morphological maturation, as evidenced by a positive force–frequency relationship, increased conduction velocity of depolarization signals and improved sarcomere orientation with distinct Z-bands compared to unstimulated controls. We confirmed that electrical stimulation enables the evaluation of the positive inotropic effects of drug compounds with diverse pharmacological actions. The functional maturation induced by the electrical stimulation was observed across different facilities. The method also effectively detected prolonged QT intervals. These findings demonstrate the utility of the electrical stimulation for 48 h for hiPSC-CMs using the multielectrode array assay system to assess drug-induced contractile function and detect prolonged QT intervals in a single experiment, thereby enhancing the early-stage assessment of cardiotoxicity in drug discovery.

## Introduction

Predicting drug safety remains challenging in drug discovery. Early assessment of safety liabilities, especially cardiotoxicity like QT prolongation and proarrhythmic risk, is essential to reduce fatal adverse effects (Tabo et al. 2010; Gintant et al. 2020; Leishman et al. 2020; Pognan et al. 2023). Additionally, evaluating effects on cardiac contractile force is important, as alterations can lead to cardiovascular events or heart failure (Wallis et al. 2015). This is especially critical in anticancer drug development, where contractility impairment can contribute to increased circulatory disorders later in life (Bhatia et al. 2023).

The Langendorff assay (Tabo et al. 2010) and left ventricular ejection fraction assessment in large animals (Guth et al. 2015) are commonly used to evaluate drug-induced changes in cardiac contractile force. While comprehensive, these methods have limitations: The Langendorff assay is low throughput, technically challenging, and unsuitable for long-term studies; ejection fraction assessments require significant expertise, leading to variability. Additionally, both approaches face species differences and conflict with the 3Rs principles (Replacement, Reduction, Refinement) (Yanagida et al. 2021), reducing their suitability for early-stage drug discovery.

Human primary cardiomyocytes or ventricular trabeculae obtained from ethically consented organ donors serve as alternative models for evaluating drug-induced changes in cardiac contractile force (Page et al. 2016; Britton et al. 2017; Qu et al. 2017; Nguyen et al. 2017; Abi-Gerges et al. 2020). Although these materials exhibit physiologically relevant phenotypes similar to native human cardiomyocytes or heart tissue, their limited availability and potential loss of cardiac function during preparation make them challenging to use in early-stage drug discovery (Guo et al. 2018).

Human induced pluripotent stem cell-derived cardiomyocytes (hiPSC-CMs), which express cardiac-specific genes and proteins, reproduce cardiomyocyte-specific action potentials and exhibit arrhythmogenic mechanisms, making them promising tools for cardiotoxicity risk assessment in early drug discovery (Ma et al. 2011). In vitro use of hiPSC-CMs has shown potential for improving drug safety evaluation, especially for QT interval prolongation and arrhythmia risk. The ICH S7B Q&A Best Practice document recognizes human cardiomyocytes, including hiPSC-CMs, as suitable models for assessing arrhythmogenic potential (Ando et al. 2017; Blinova et al. 2018; Gintant et al. 2020; International Council for Harmonisation 2022). However, hiPSC-CMs cultured using current two-dimensional methods remain functionally immature, with underdeveloped sarcomere architecture, impaired gap-junction communication, deficient Ca^2+^ handling, altered metabolism, and lower resting membrane potentials compared to adult cardiomyocytes (Germanguz et al. 2011; Yang et al. 2023; Li et al. 2024). These limitations restrict accurate evaluation of pharmacological effects on cardiac contractility (Scott et al. 2014; Okai et al. 2020).

Various methods have been explored to promote maturity of hiPSC-CMs, including cell alignment (Satsuka et al. 2024), coculture with fibroblasts and vascular endothelial cells (Beauchamp et al. 2020), addition of bioactive agents (Breckwoldt et al. 2017; Parikh et al. 2017), and formation of three-dimensional tissue constructs (Breckwoldt et al. 2017; Feric et al. 2019; Li et al. 2024). Although these approaches enhance cardiomyocyte maturation, they often involve complex and costly protocols, limiting their practical application in early-stage drug discovery.

Functional maturity of hiPSC-CMs can be promoted by electrical stimulation using specialized cell culture plates with electrodes placed on the bottom surface (Zhang et al. 2023). This method offers a considerable advantage at an early stage of drug discovery, as it promotes the development of cardiac contractile force and enables the measurement of cardiac function and impedance in the same culture format. However, the protocol previously reported requires long-term (15-d) continuous electrostimulation (Zhang et al. 2023), posing barriers for early-stage drug discovery, considering its duration and equipment occupancy.

To address the limitations of current in vitro models for assessing cardiac contractility, the present study aimed to establish a rapid, high-throughput, and robust method suitable for early-stage drug discovery using multielectrode array (MEA) system. We investigated the effectiveness of a short-term electrical stimulation protocol in enhancing the functional properties of hiPSC-CMs and in evaluating pharmacological effects on cardiac contractility. Additionally, we examined whether this method could detect both QT interval prolongation and proarrhythmic effects. Our approach involved electrical stimulation of hiPSC-CMs to induce a more mature phenotype, assessing parameters such as the force–frequency relationship (FFR), conduction velocity of the depolarization signals through the cultured cardiomyocytes, and pharmacological responsiveness. We also evaluated other maturity markers, including ultrastructure, Ca^2+^ handling, and gene expression profiles. Furthermore, we tested whether these findings could be replicated across two independent laboratories.

## Materials and methods

### Cell culture

iCell cardiomyocytes^2^ (iCell-CM^2^; Fujifilm Cellular Dynamics, Madison, WI, USA) were obtained and prepared according to the manufacturer’s instructions. A Cytoview MEA 24-well plate (Axion Biosystems, Atlanta, GA, USA) was coated with a 50 µg/mL aqueous solution of human plasma-derived fibronectin (Roche, Basel, Switzerland) and incubated at 37°C for 1 h. Subsequently, iCell-CM^2^ suspended in iCell CM plating medium (Fujifilm Cellular Dynamics) were seeded at a density of 40,000 to 50,000 cells per well. The plate was incubated in a humidified CO_2_ incubator at 37°C for 1 h to allow cell adhesion, after which iCell CM maintenance medium (Fujifilm Cellular Dynamics) was added and the cells were cultured for 7 d. The media were changed every 2–3 d during the culture period.

### Electrical stimulation of hiPSC-CMs in the MEA system

**Fig. 1.**
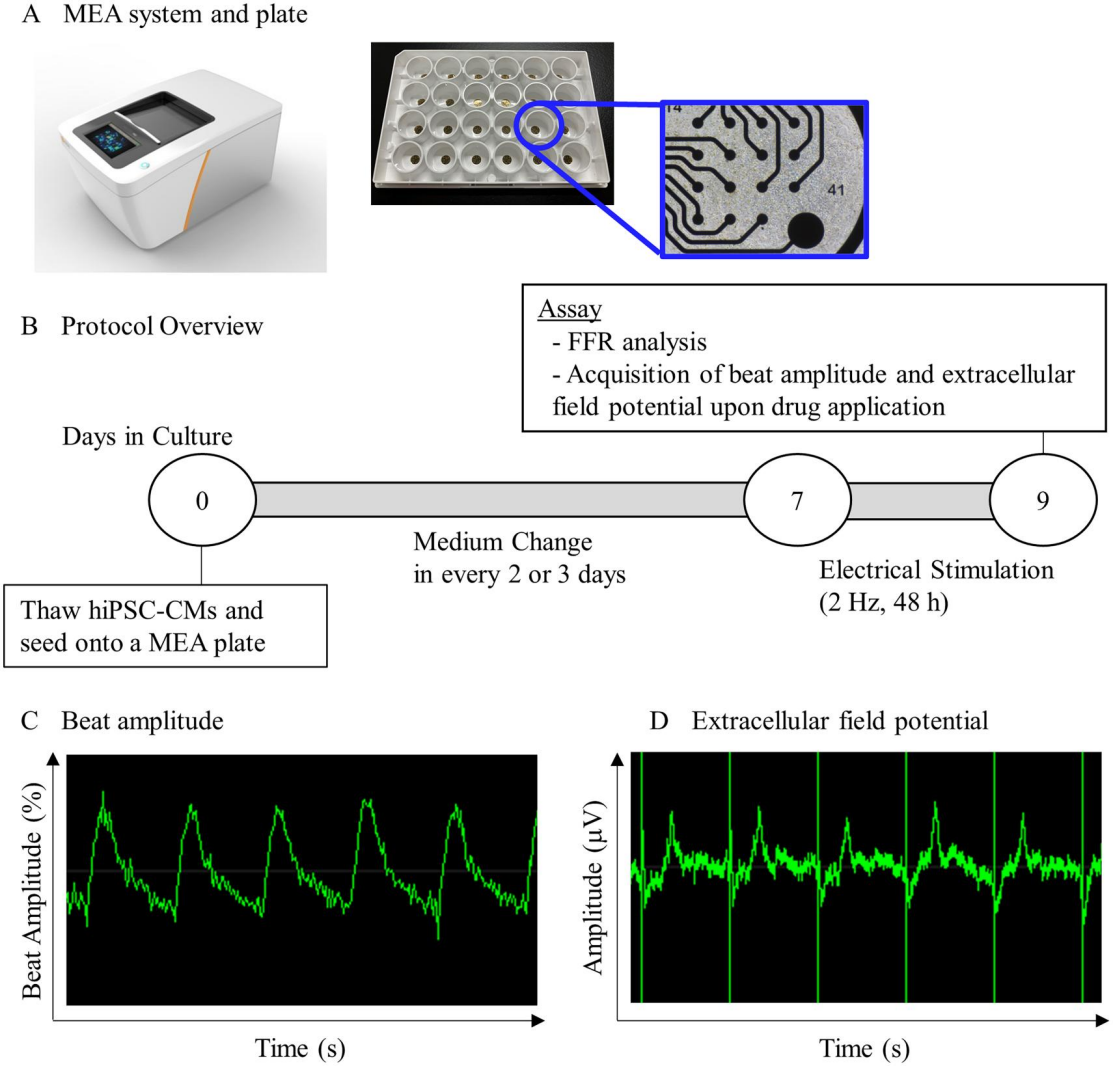
A) Representative image of the multielectrode array (MEA) system and plate. B) Protocol overview for cell culture and assay. Human induced pluripotent stem cell-derived cardiomyocytes (hiPSC-CMs) were cultured in a MEA plate and electrical stimulation was initiated at 7th day of culture and continued for 48 h, followed by evaluation of the force–frequency relationship (FFR) and drug responses. Beat amplitude C) and extracellular field potential D) detected by MEA system were used as indicators to evaluate cardiac contractility and cardiac action potential.

After culturing the cells for 7 d, the MEA plate containing the cells was transferred from the CO_2_ incubator to the chamber of the MEA system (Maestro Pro; Axion Biosystems), where the temperature was maintained at 37°C with 5% CO_2_. The cells were electrically stimulated for 48 h using a pacing amplitude of 40 µV and a frequency of 2 Hz via the stimulating electrode on the MEA plate, following the manufacturer’s application note for the MEA system. An overview of the experimental timeline is presented in Fig. 1A and B.

### Evaluation of the force–frequency relationship

After completion of the 48 h electrical stimulation, the beat amplitude, a measurement of movement and hence cardiomyocyte contractility, was assessed using the MEA system and its software (Axis Navigator; Axion Biosystems). The beat amplitude was measured for 1 min during spontaneous beating in the absence of electrical stimulation. Subsequently, beat amplitude was measured while incrementally increasing the stimulation frequency every minute to 1.0, 1.25, 1.5, 2.0, and 2.5 Hz at Facility A, or 1.0, 1.5, 2.0, 2.5, and 3.0 Hz at Facility B. To evaluate the FFR, we examined the relationship between the frequency of electric stimulation and the percentage change in beat amplitude against spontaneous beating. Representative images of the beat amplitude and extracellular field potential acquired in this study are shown in Fig. 1C and D.

### Evaluation of conduction velocity

After the 48 h electrical stimulation period in the MEA system, the conduction velocity of the signals propagating through the cells was measured using Axis Navigator software (Axion Biosystems) during spontaneous beating, with 0.1% dimethyl sulfoxide (DMSO) added to the cells as Dose 0 for a subsequent drug response assay. The representative conduction velocity for each condition was evaluated by selecting wells where the signal originated uniquely and propagated in a unidirectional, fan-shaped manner.

### Evaluation of intracellular Ca^2+^ concentration of hiPSC-CMs

hiPSC-CMs were cultured in an MEA plate for 10 d after the cells were seeded. Electrical stimulation was conducted from day 8 to 10. On day 10, the media was renewed, followed by 2 h incubation in a CO_2_ incubator. Then, half of the media was changed with the EarlyTox Cardiotoxicity Kit (Molecular Devices, R8210), and the cells were incubated for a further 2 h in a CO_2_ incubator. Subsequently, the cells were put into the FDSS/µCELL system (Hamamatsu Photonics, Shizuoka, Japan). The fluorescent Ca^2+^ signals were measured using the FDSS/µCELL platform, and the records were later analyzed offline using U8524-12 software (Hamamatsu Photonics) to detect beat-by-beat amplitude. All wells within a plate were measured simultaneously using the following FDSS/µCELL settings: Sampling frequency, 30 ms; excitation wavelength, 480 nm; emission wavelength, 540 nm; with temperature controlled at 37°C.

### Gene expression analysis

Total RNA was extracted from hiPSC-CMs cultured in MEA plates on day 4, 7, 9, 11, and 14. Electrical stimulation was conducted from day 7 to 11. An RNeasy Mini kit (Qiagen, 74104) was used according to the manufacturer’s instructions to prepare cell lysate and to extract and purify total RNA. Total RNA was extracted and purified from four samples each day, both with and without electrical stimulation, and then pooled together. Total RNA isolated from adult human heart tissue (Clontech, 636532)—prepared using a modified guanidinium thiocyanate method from normal heart samples pooled from three male Caucasians aged 30 to 39—was obtained from Takara Bio (Shiga, Japan).

The pooled RNA samples were mixed with reagents from the QuantiTect SYBR Green RT-PCR kit (Qiagen, 204243) and primers purchased from Fasmac (Kanagawa, Japan) (HA067812 for GAPDH, HA010114 for TNNI3, HA347602 for RYR2, HA398587 for APT2A2, HA329084 for PDE3A, and HA143939 for PDE3B). PCR analysis was performed in duplicate using a Bio-Rad CFX384 system.

### Transmission electron microscopy

The microstructure of the cultured iCell-CM^2^ was examined using transmission electron microscopy with an accelerating voltage of 100 kV. To prepare the samples for observation, the iCell-CM^2^ cultured in the MEA plate were fixed in a 2% glutaraldehyde solution for 1 h. Subsequently, they were treated with a 1% osmium tetroxide solution for 1 h, dehydrated, embedded in epoxy resin, and cut into ultrathin (100 nm) sections. The sections were then stained with an electron microscopy stainer (Nisshin-EM, Tokyo, Japan) and lead citrate for further analysis.

### Test compounds

DMSO, forskolin, milrinone, verapamil, E-4031, dobutamine, and propranolol were purchased from Fujifilm Wako Pure Chemical (Osaka, Japan). Isoproterenol, digoxin, pimobendan, and mexiletine were purchased from Tokyo Chemical Industry Co. (Tokyo, Japan). (*S*)-(−)-BayK8644 was purchased from Tocris Biosciences (Bristol, UK). Omecamtiv mecarbil was purchased from Selleck Chem (Houston, TX, USA). The compounds were dissolved in DMSO. The compounds have known effects on cardiac contractility, QT interval, or arrhythmogenicity (Scott et al. 2014; Guth et al. 2015; Nozaki et al. 2016). Pharmacological actions of the compounds are presented in Tables 1,  2 and 3.

**Table 1.** Effect of electrical stimulation on responses of beat amplitude on reference compounds.

Compounds	Pharmacological action	Concentration					Facility A			
				Without electrical stimulation			With electrical stimulation			
				Beat amplitude % change (absolute value)	Beat stop	*n*	Beat Amplitude % change (absolute value)	Beat stop	*n*	*P* value
Positive inotropes										
Isoproterenol	nonselective β adrenergic receptor agonist	0	µM	**0.0 ± 0.0** (1.65 ± 0.05)	–	4	**0.0 ± 0.0** (1.06 ± 0.06)	–	3	**n/a**
		0.3		**−0.4 ± 5.8** (1.64 ± 0.10)	–		**21.7 ± 12.4** (1.20 ± 0.06)	–		**ns**
		1		**−1.8 ± 5.1** (1.62 ± 0.07)	–		**39.2 ± 14.3** (1.33 ± 0.07)	–		**<0.05**
		3		**−9.3 ± 4.9** (1.49 ± 0.07)	–		**35.4 ± 11.3** (1.31 ± 0.05)	–		**<0.01**
		10		**−7.5 ± 4.5** (1.52 ± 0.07)	–		**45.5 ± 12.8** (1.42 ± 0.06)	–		**<0.01**
Forskolin	Adenylyl cyclase activator	0	µM	**0.0 ± 0.0** (1.83 ± 0.15)	–	4	**0.0 ± 0.0** (1.34 ± 0.19)	–	3	**n/a**
		0.1		**−4.0 ± 3.7** (1.74 ± 0.09)	–		**10.5 ± 5.0** (1.49 ± 0.23)	–		**ns**
		0.3		**−1.1 ± 3.7** (1.79 ± 0.10)	–		**19.7 ± 10.8** (1.58 ± 0.26)	–		**ns**
		1		**−3.8 ± 5.1** (1.74 ± 0.07)	–		**25.5 ± 7.3** (1.65 ± 0.23)	–		**<0.05**
		3		**−7.3 ± 6.9** (1.66 ± 0.05)	–		**28.2 ± 4.7** (1.69 ± 0.25)	–		**<0.05**
(S)-(−)-BayK8644	L-type Ca^2+^ channel activator	0	µM	**0.0 ± 0.0** (2.02 ± 0.11)	–	4	**0.0 ± 0.0** (1.50 ± 0.27)	–	4	**n/a**
		0.01		**−2.9 ± 1.0** (1.96 ± 0.09)	–		**12.4 ± 9.1** (1.72 ± 0.39)	–		**ns**
		0.03		**7.6 ± 2.1** (2.17 ± 0.09)	–		**26.7 ± 10.8** (1.90 ± 0.36)	–		**ns**
		0.1		**6.7 ± 4.5** (2.16 ± 0.17)	–		**39.2 ± 8.0** (2.09 ± 0.40)	–		**<0.05**
		0.3		**7.7 ± 4.3** (2.18 ± 0.17)	–		**50.6 ± 6.0** (2.27 ± 0.44)	–		**<0.01**
Digoxin	Na+/K+ ATPase inhibitor	0	µM	**0.0 ± 0.0** (1.86 ± 0.11)	–	4	**0.0 ± 0.0** (1.25 ± 0.13)	–	4	**n/a**
		0.03		**2.7 ± 3.7** (1.90 ± 0.06)	–		**5.3 ± 5.0** (1.33 ± 0.18)	–		**ns**
		0.1		**4.9 ± 2.5** (1.94 ± 0.07)	–		**17.9 ± 7.0** (1.48 ± 0.17)	–		**ns**
		0.3		**0.8 ± 2.9** (1.87 ± 0.11)	–		**37.2 ± 9.7** (1.70 ± 0.15)	–		**<0.05**
		1		**−1.5** (1.86)	3/4		**55.3 ± 19.1** (2.10 ± 0.40)	1/4		**ns**
Negative inotropes										
Verapamil	L-type Ca^2+^ channel blocker	0	µM	**0.0 ± 0.0** (2.29 ± 0.22)	–	4	**0.0 ± 0.0** (1.42 ± 0.14)	–	2	**n/a**
		0.03		**−17.8 ± 2.9** (1.89 ± 0.22)	–		**−28.1 ± 6.4** (1.04 ± 0.18)	–		**ns**
		0.1		**−21.4 ± 5.8** (1.77 ± 0.12)	–		**−43.6 ± 1.0** (0.86 ± 0.07)	–		**ns**
		0.3		**−53.7 ± 9.5** (1.00 ± 0.13)	–		**−50.2 ± 7.8** (0.67 ± 0.03)	–		**ns**
		1		**−72.2 ± 3.9** (0.61 ± 0.05)	–		**−54.7 ± 8.1** (0.57 ± 0.04)	–		**ns**
Vehicle control										
DMSO		Dose0		**0.0 ± 0.0** (1.94 ± 0.03)	–	4	**0.0 ± 0.0** (1.27 ± 0.04)	–	20	**n/a**
		Dose1		**−0.9 ± 3.3** (1.92 ± 0.07)	–		**−1.1 ± 1.0** (1.25 ± 0.04)	–		**ns**
		Dose2		**3.5 ± 5.1** (2.01 ± 0.12)	–		**1.1 ± 1.4** (1.28 ± 0.05)	–		**ns**
		Dose3		**0.2 ± 6.8** (1.94 ± 0.13)	–		**2.4 ± 1.7** (1.27 ± 0.04)	–		**ns**
		Dose4		**−0.4 ± 5.4** (1.93 ± 0.11)	–		**2.9 ± 1.8** (1.35 ± 0.04)	–		**ns**

Beat amplitude (% change) is shown as mean ± standard error of the mean.

Significant differences between the electrically stimulated and non-stimulated groups were evaluated using a paired Student’s t-test with a significance threshold of *P *= 0.05.

DMSO, dimethyl sulfoxide; n/a; not available; ns, not significant.

**Table 2.** Responses of beat amplitude to reference compounds.

Compounds	Pharmacological action	Concentration		Facility A				Facility B			
				Beat amplitude % change (absolute value)	Beat stop	*n*	*P* value	Beat amplitude % change (absolute value)	Beat stop	*n*	*P* value
Positive inotropes											
Isoproterenol	nonselective β adrenergic receptor agonist	0	µM	**0.0 ± 0.0** (1.06 ± 0.06)	–	3	**n/a**	**0.0 ± 0.0** (4.01 ± 2.01)		3	**n/a**
		0.3		**21.7 ± 12.4** (1.20 ± 0.06)	–		**<0.01**	**105.5 ± 102.2** (5.06 ± 0.72)	–		**ns**
		1		**39.2 ± 14.3** (1.33 ± 0.07)	–		**<0.005**	**129.3 ± 104.3** (5.63 ± 0.41)	–		**ns**
		3		**35.4 ± 11.3** (1.31 ± 0.05)	–		**<0.05**	**159.3 ± 128.0** (6.15 ± 0.67)	–		**ns**
		10		**45.5 ± 12.8** (1.42 ± 0.06)	–		**<0.001**	**189.6 ± 135.0** (7.23 ± 0.81)	–		**ns**
Dobutamine	β-1 adrenergic receptor agonist	0	µM	**0.0 ± 0.0** (2.80 ± 0.70)	–	3	**n/a**	**NT**			
		0.01		**10.9 ± 12.9** (3.28 ± 1.20)	–		**ns**				
		0.1		**19.1 ± 15.4** (3.26 ± 0.83)	–		**ns**				
		1		**40.8 ± 27.8** (3.78 ± 0.96)	–		**<0.005**				
		10		**−10.9 ± 7.9** (2.49 ± 0.68)	–		**ns**				
Forskolin	Adenylyl cyclase activator	0	µM	**0.0 ± 0.0** (1.34 ± 0.19)	–	3	**n/a**	**NT**			
		0.1		**10.5 ± 5.0** (1.49 ± 0.23)	–		**ns**				
		0.3		**19.7 ± 10.8** (1.58 ± 0.26)	–		**ns**				
		1		**25.5 ± 7.3** (1.65 ± 0.23)	–		**ns**				
		3		**28.2 ± 4.7** (1.69 ± 0.25)	–		**<0.05**				
								**NT**			
(S)-(-)-BayK8644	L-type Ca^2+^ channel activator	0	µM	**0.0 ± 0.0** (1.50 ± 0.27)			**n/a**				
		0.01		**12.4 ± 9.1** (1.72 ± 0.39)	–	4	**ns**				
		0.03		**26.7 ± 10.8** (1.90 ± 0.36)	–		**<0.01**				
		0.1		**39.2 ± 8.0** (2.09 ± 0.40)	–		**<0.001**				
		0.3		**50.6 ± 6.0** (2.27 ± 0.44)	–		**<0.001**				
Digoxin	Na^+^/K^+^ ATPase inhibitor	0	µM	**0.0 ± 0.0** (1.25 ± 0.13)	–	4	**n/a**	**NT**			
		0.03		**5.3 ± 5.0** (1.33 ± 0.18)	–		**ns**				
		0.1		**17.9 ± 7.0** (1.48 ± 0.17)	–		**ns**				
		0.3		**37.2 ± 9.7** (1.70 ± 0.15)	–		**<0.005**				
		1		**55.3 ± 19.1** (2.10 ± 0.40)	1/4		**<0.001**				
Ouabain	Na^+^/K^+^ ATPase inhibitor	0	µM	**NT**				**0.0 ± 0.0** (2.72 ± 0.55)		3	**n/a**
		0.003						**13.2 ± 16.7** (3.18 ± 0.96)	–		**ns**
		0.01						**126.4 ± 59.0** (5.54 ± 0.74)	–		**ns**
		0.03						**132.9 ± 87.2** (5.40 ± 1.59)	–		**ns**
		0.1						**260.3 ± 268.7** (8.81 ± 5.31)	1/3		**ns**
Milrinone	PDE-3 inhibitor	0	µM	**0.0 ± 0.0** (1.31 ± 0.05)	–	6	**n/a**	**0.0 ± 0.0** (3.49 ± 1.03)		3	**n/a**
		1		**1.5 ± 3.8** (1.36 ± 0.07)	–		**ns**	**49.6 ± 14.0** (4.95 ± 1.02)	–		**ns**
		3		**2.9 ± 3.9** (1.38 ± 0.07)	–		**ns**	**47.2 ± 8.9** (4.99 ± 1.26)	–		**ns**
		10		**10.8 ± 4.0** (1.47 ± 0.06)	–		**ns**	**93.6 ± 45.9** (5.92 ± 0.52)	–		**ns**
		30		**11.8 ± 5.2** (1.52 ± 0.08)	–		**ns**	**93.1 ± 67.1** (5.46 ± 0.49)	–		**ns**
Pimobendan	calcium sensitizer with PDE-3 inhibition	0	µM	**0.0 ± 0.0** (0.99 ± 0.04)	–	4	**n/a**	**0.0 ± 0.0** (1.39 ± 0.19)		3	**n/a**
		0.3		**0.7 ± 3.1** (0.99 ± 0.04)	–		**ns**	**38.0 ± 16.8** (1.94 ± 0.42)	–		**ns**
		1		**3.9 ± 4.7** (1.02 ± 0.06)	–		**ns**	**76.4 ± 29.2** (2.43 ± 0.41)	–		**ns**
		3		**2.7 ± 3.8** (1.01 ± 0.04)	–		**ns**	**116.5 ± 17.6** (2.94 ± 0.15)	–		**ns**
		10		**11.4 ± 4.2** (1.10 ± 0.05)	–		**ns**	**172.7 ± 17.7** (3.72 ± 0.25)	–		**ns**
Omecamtiv Mecarbil	Myosin activator	0	µM	**0.0 ± 0.0** (1.48 ± 0.02)	–	3	**n/a**	**NT**			
		0.1		**2.4 ± 2.5** (1.50 ± 0.05)	–		**ns**				
		0.3		**8.1 ± 2.7** (1.54 ± 0.04)	–		**ns**				
		1		**21.4 ± 6.4** (1.73 ± 0.09)	–		**ns**				
		3		**15.0 ± 0.5** (1.63 ± 0.03)	–		**ns**				
Negative inotropes											
Verapamil	L-type Ca^2+^ channel blocker	0	µM	**0.0 ± 0.0** (1.42 ± 0.14)	–	2	**n/a**	**NT**			
		0.03		**−28.1 ± 6.4** (1.04 ± 0.18)	–		**<0.01**				
		0.1		**−43.6 ± 1.0** (0.86 ± 0.07)	–		**<0.005**				
		0.3		**−50.2 ± 7.8** (0.67 ± 0.03)	–		**<0.001**				
		1		**−54.7 ± 8.1** (0.57 ± 0.04)	–		**<0.001**				
Mexiletine	Na^+^ channel blocker	0	µM	**0.0 ± 0.0** (1.57 ± 0.13)	–	3	**n/a**	**NT**			
		1		**−0.1 ± 5.0** (1.67 ± 0.19)	–		**ns**				
		3		**3.9 ± 3.6** (1.72 ± 0.19)	–		**ns**				
		10		**−5.6 ± 5.5** (1.55 ± 0.22)	–		**ns**				
		30		**−23.5** (1.13)	2/3		**ns**				
Propranolol	nonselective β receptor agonist	0	µM	**0.0 ± 0.0** (1.55 ± 0.12)	–	4	**n/a**	**NT**			
		0.3		**2.3 ± 4.3** (1.58 ± 0.08)	–		**ns**				
		1		**5.3 ± 2.6** (1.63 ± 0.09)	–		**ns**				
		3		**−1.5 ± 7.6** (1.51 ± 0.09)	–		**ns**				
		10		**−11.0 ± 2.1** (1.21 ± 0.04)	2/4		**ns**				
Vehicle control											
DMSO		Dose0		**0.0 ± 0.0** (1.27 ± 0.04)	–	20	**n/a**	**0.0 ± 0.0** (3.46 ± 0.43)		3	**n/a**
		Dose1		**−1.1 ± 1.0** (1.25 ± 0.04)	–		**n/a**	**13.2 ± 11.1** (3.73 ± 0.71)	–		**n/a**
		Dose2		**1.1 ± 1.4** (1.28 ± 0.05)	–		**n/a**	**6.8 ± 10.8** (3.86 ± 0.73)	–		**n/a**
		Dose3		**2.4 ± 1.7** (1.27 ± 0.04)	–		**n/a**	**15.3 ± 7.6** (4.18 ± 0.63)	–		**n/a**
		Dose4		**2.9 ± 1.7** (1.35 ± 0.04)	–		**n/a**	**31.1 ± 8.6** (4.20 ± 0.61)	–		**n/a**

Beat amplitude (% change) is shown as mean ± standard error of the mean.

Significant differences from the vehicle control were evaluated using one-way ANOVA followed by Dunnett’s post hoc test, with a significance threshold of *P *= 0.05.

DMSO, dimethyl sulfoxide; n/a; not available; ns, not significant; NT, not tested.

**Table 3.** Field potential responses to reference compounds.

Compounds		Pharmacological Action	Concentration		Facility A				
					FPDc % change (FPDc [ms])	EAD	Abnormal	n	*p* value
Test compounds								
E-4031	hERG inhibitor	0	nM	**0.0 ± 0.0** (324 ± 18)			3	**n/a**
		1.6		**10.0 ± 1.1** (356 ± 26)	–	–		**ns**
		5		**20.0 ± 1.1** (389 ± 28)	–	–		**ns**
		16		**36.5 ± 4.2** (442 ± 49)	–	–		**<0.005**
		50		**N.A.** (N.A.)	1/3	1/3		**n/a**
Verapamil	L-type Ca^2+^ channel blocker	0	µM	**0.0 ± 0.0** (317 ± 23)			3	**n/a**
		0.03		**−10.8 ± 10.9** (283 ± 42)	–	–		**ns**
		0.1		**−20.7 ± 11.1** (251 ± 45)	–	–		**<0.005**
		0.3		**−41.0 ± 8.9** (187 ± 38)	–	–		**<0.001**
		1		**−55.8 ± 6.7** (139 ± 30)	–	–		**<0.001**
Isoproterenol	nonselective β adrenergic receptor agonist	0	µM	**0.0 ± 0.0** (309 ± 32)			4	**n/a**
		0.3		**3.0 ± 2.7** (318 ± 29)	–	–		**ns**
		1		**5.3 ± 3.0** (325 ± 38)	–	–		**ns**
		3		**−0.8 ± 2.8** (306 ± 21)	–	–		**ns**
		10		**−2.5 ± 4.6** (301 ± 7)	–	–		**ns**
Vehicle control								
DMSO		Dose0		**0.0 ± 0.0** (343 ± 24)			4	**-**
		Dose1		**3.3 ± 2.4** (354 ± 30)	–	–		**n/a**
		Dose2		**7.8 ± 1.9** (370 ± 33)	–	–		**n/a**
		Dose3		**9.7 ± 1.5** (376 ± 23)	–	–		**n/a**
		Dose4		**10.5 ± 2.2** (379 ± 17)	–	–		**n/a**

Field potential duration corrected for beat rate (i.e., the spontaneous or paced heart rate of the cardiomyocytes) (FPDc) is shown as mean ± standard error of the mean.

Significant differences from the vehicle control were evaluated using one-way ANOVA followed by Dunnett’s post hoc test, with a significance threshold of *P *= 0.05.

DMSO, dimethyl sulfoxide; EAD; early afterdepolarization; n/a; not available; ns, not significant.

### Drug response

The DMSO solutions of the compounds were diluted using iCell CMs maintenance medium to achieve a final DMSO concentration of 0.1%. Subsequently, the solution of each compound was cumulatively added to each well of the MEA plate, and the beat amplitude or extracellular field potential was measured at each concentration. To analyze the beat amplitude, the mean value of all electrodes (Facility A) or the mean value of electrodes after omission of those that did not detect the cardiac beat clearly (Facility B) was used. For the analysis of extracellular field potentials, a single electrode exhibiting a typical extracellular field potential of hiPSC-CMs was chosen based on previous reports (Kanda et al. 2016).

### Statistical analysis

**Fig. 2.**
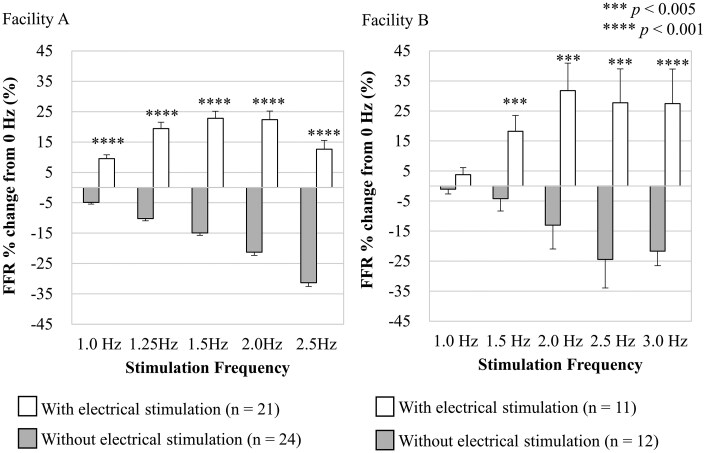
Evaluation of functional maturation of human induced pluripotent stem cell-derived cardiomyocytes (hiPSC-CMs) using force–frequency relationship (FFR). Effect of electrical stimulation on FFR of hiPSC-CMs were evaluated at two different facilities. The graphs show the mean values for each stimulation frequency after cells were cultured with or without electrical stimulation. The error bars represent the standard error. The number of replicates per group is indicated in the figure: Facility A (*n* = 21 with electrical stimulation, *n* = 24 without) and Facility B (*n* = 11 with electrical stimulation, *n* = 12 without). A Student *t* test was applied to investigate if significant differences were observed between the group with or without electrical stimulation at each dose. The *P* values are indicated in the figure as follows: **P < 0.01; ***P < 0.005; ****P < 0.001.

**Fig. 3.**
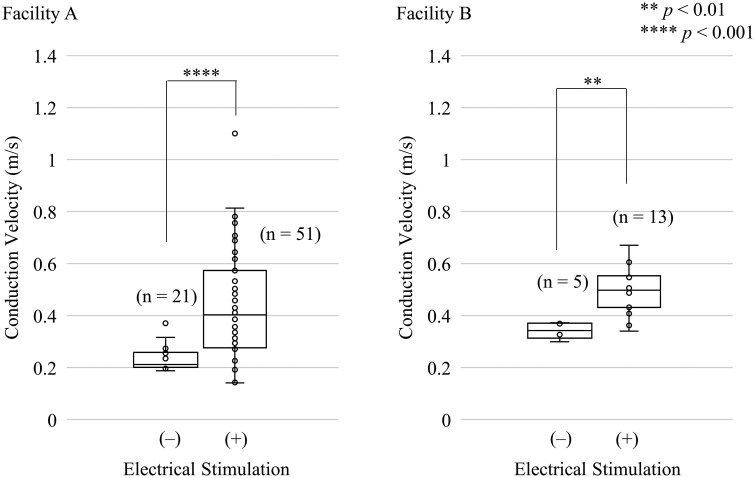
Evaluation of conduction velocity of human induced pluripotent stem cell-derived cardiomyocytes (hiPSC-CMs) with (+) or without (–) electrical stimulation analyzed at both facilities. The data are visualized in a box plot. In the box plot, the box represents the interquartile range (IQR, 25th to 75th percentiles), the line inside the box indicates the median, and the whiskers extend to the minimum and maximum values within 1.5 times the IQR. Outliers beyond this range are shown as individual points. The number of replicates per group is indicated in the figure: Facility A (*n* = 51 with electrical stimulation, *n* = 21 without) and Facility B (*n* = 13 with electrical stimulation, *n* = 5 without). A Student *t* test was applied to determine if significant differences were observed between the group with or without electrical stimulation at each dose. The *P* values are indicated in the figure as follows: **P < 0.01; ****P < 0.001.

**Fig. 4.**
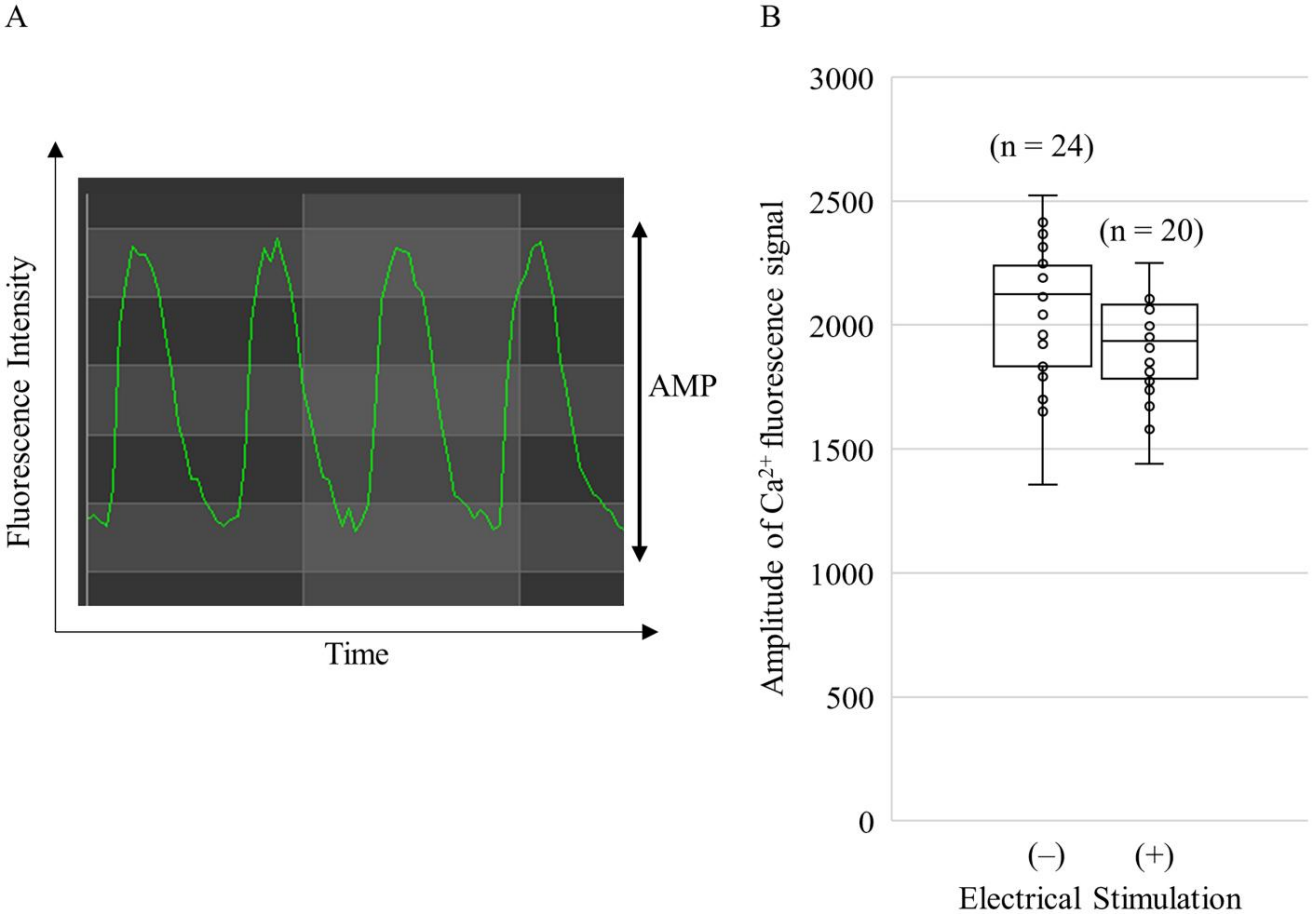
Intracellular Ca^2+^ concentration of human induced pluripotent stem cell-derived cardiomyocytes (hiPSC-CMs). A) Image for analysis of intracellular Ca^2+^ concentration visualized by a fluorescent Ca^2+^ indicator. B) Amplitude (AMP) of the Ca^2+^ fluorescence signal of hiPSC-CMs with or without electrical stimulation. The data are visualized in a box plot. In the box plot, the box represents the interquartile range (IQR, 25th to 75th percentiles), the line inside the box indicates the median, and the whiskers extend to the minimum and maximum values within 1.5 times the IQR. Outliers beyond this range are shown as individual points. The number of replicates per group is indicated in the figure (*n* = 20 with electrical stimulation, *n* = 24 without).

**Fig. 5.**
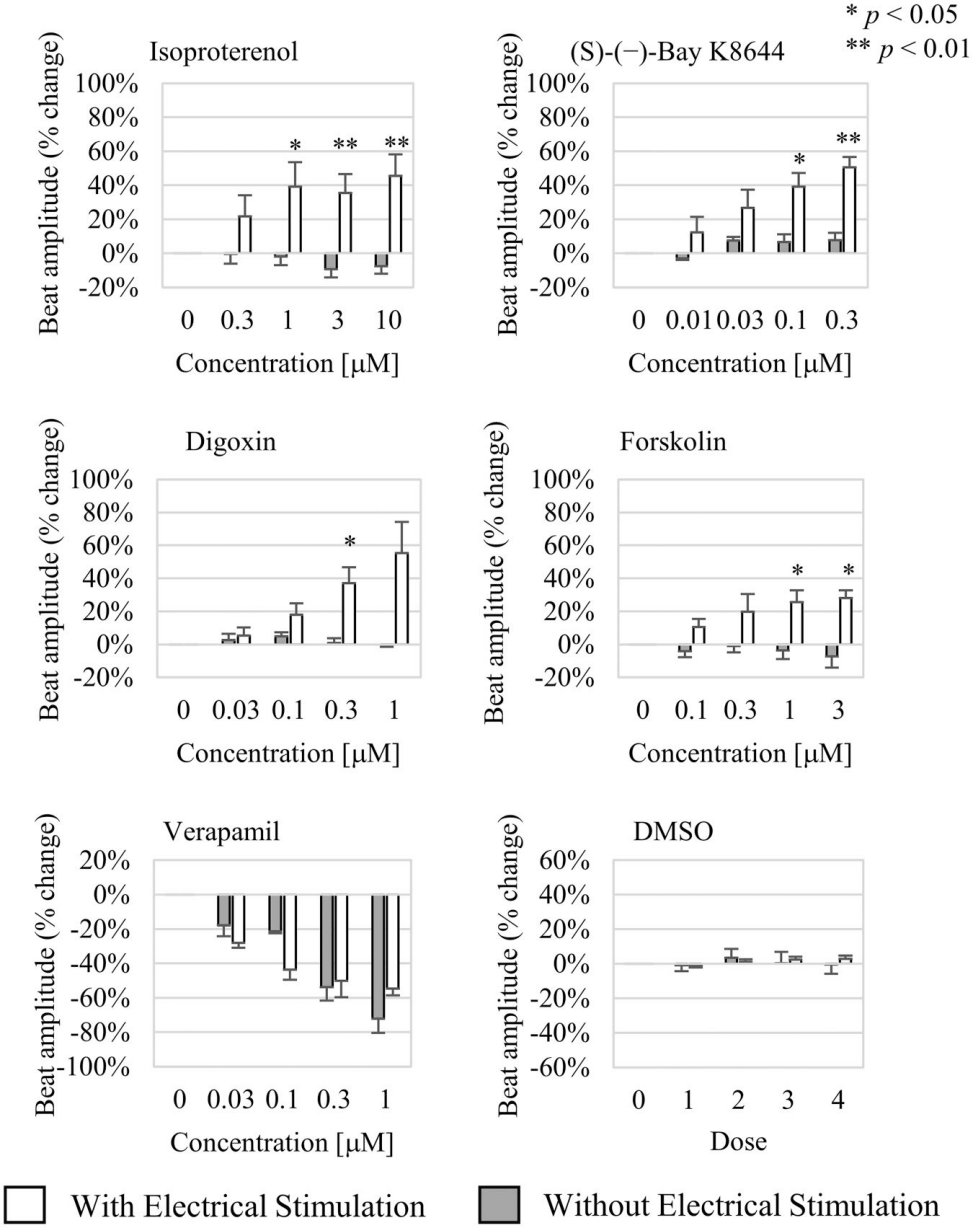
Responses to various drugs with or without electrical stimulation. The bars show the mean change in beat amplitude relative to the initial value before drug exposure with or without electrical stimulation. The error bars represent standard errors of the mean. Exact values, the number of replicates (*n* = 2 to 20), and the significance of differences between groups with or without electrical stimulation are provided in Table 1. A Student *t* test was used to determine significant differences between the group with or without electrical stimulation at each dose. The *P* values are indicated in the figure as follows: *P < 0.05, **P < 0.01. DMSO, dimethyl sulfoxide.

**Fig. 6.**
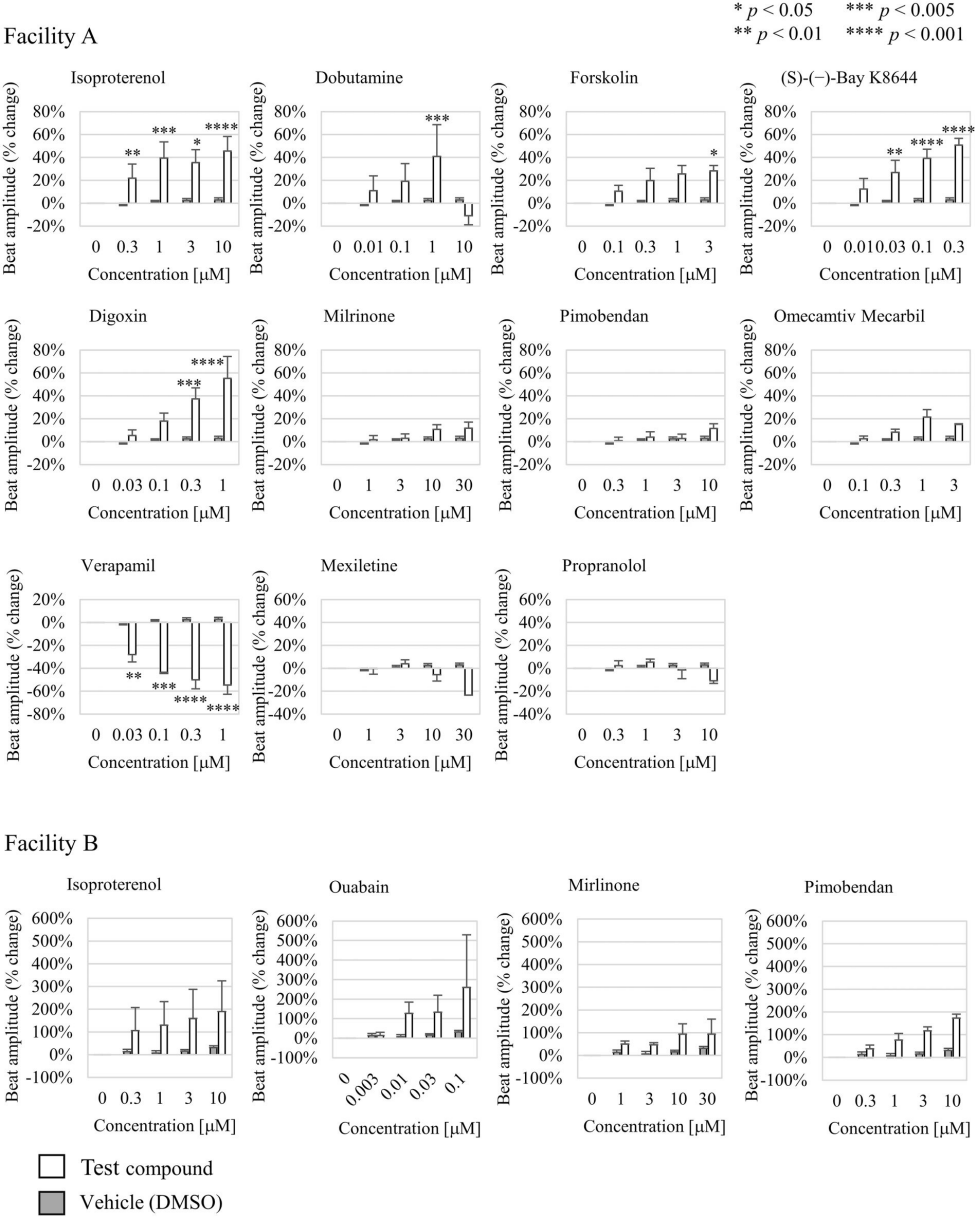
Responses to various drugs tested at the two different facilities. The bars represent results of vehicle (dimethyl sulfoxide, DMSO) control (pale gray) and each compound (filled in black). The error bars represent the standard errors of the means. Exact values, the number of replicates (*n* = 2 to 20), and the significance of differences compared to the vehicle control at each facility are provided in Table 2. One-way ANOVA followed by a Dunnett post hoc test was used to determine significant differences. The *P* values are indicated in the figure as follows: *P < 0.05, **P < 0.01, ***P < 0.005, ****P < 0.001.

**Fig. 7.**
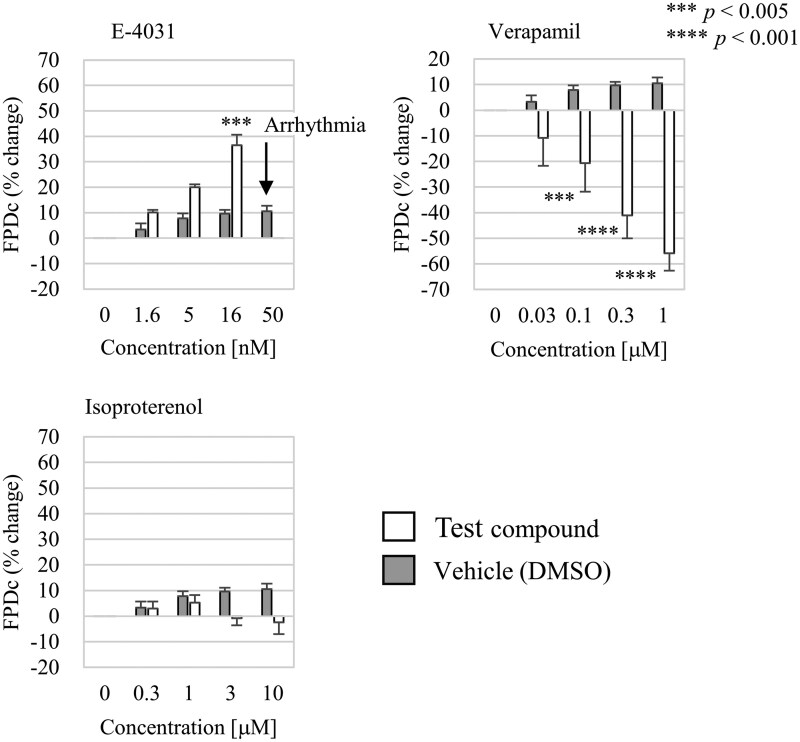
Corrected field potential duration (FPDc) change induced by test compounds. The bars represent results of vehicle (dimethyl sulfoxide, DMSO) control (pale gray) and each compound (filled in black). Exact values, the number of replicates (*n* = 3 or 4), and the significance of differences compared to the vehicle control are provided in Table 3. The error bars represent the standard error. One-way ANOVA followed by a Dunnett post hoc test was used to determine significant differences. The *P* values are indicated in the figure as follows: **P < 0.01, ****P < 0.001.

A paired Student’s *t*-test was used to assess significant differences between groups with and without electrical stimulation for the force-frequency relationship (FFR) at each stimulation frequency (Fig. 2), conduction velocity (Fig. 3), amplitude of the Ca^2+^ fluorescence signal (Fig. 4), and drug responses (Fig. 5). One-way ANOVA followed by a Dunnett post hoc test was used to determine significant differences for drug responses compared with the vehicle control (Figs. 6 and 7). Significant differences were evaluated with a significance threshold of *p *= 0.05. Microsoft Excel was used to conduct the Student *t* test and GraphPad Prism was used for the ANOVA.

## Results

### Functional maturation by electrical stimulation

To determine the functional maturity of hiPSC-CMs induced by the electrical stimulation for 48 h, we assessed the FFR of the cells by measuring the beat rate dependency of beat amplitude (impedance) on the MEA. In the absence of electrical stimulation, we observed a decrease in beat amplitude with increasing beat rate (negative FFR), which indicates an immature phenotype of the cardiomyocytes. Conversely, in the group electrically stimulated for 48 h, we observed an increase in beat amplitude with increasing beat rate up to 2.0 Hz (positive FFR), suggesting a degree of functional maturity. However, when the beat rate was equal to or exceeded 2.5 Hz, the beat amplitude decreased compared with that at 2.0 Hz. The positive force–frequency relationship (FFR) up to 2.0 Hz induced by electrical stimulation was observed in two different laboratories. However, differing trends were observed beyond 2.5 Hz between the two facilities. At facility A, the FFR percentage change from 0 Hz decreased to nearly half at 2.5 Hz compared to 2.0 Hz, whereas facility B showed a less pronounced decrease in FFR percentage change beyond 2.0 Hz.

Additionally, it was observed that the conduction velocity within the obtained cell sheets of cardiomyocytes increased with electrical stimulation. Similar trends were observed independently in two separate laboratories (Fig. 3). No significant difference in the increase of intracellular Ca^2+^ concentration upon excitation was observed between the groups without and with electrical stimulation (p = 0.07). Data are shown in Fig. 4.

### The effect of electrical stimulation on gene expression levels

**Fig. 8.**
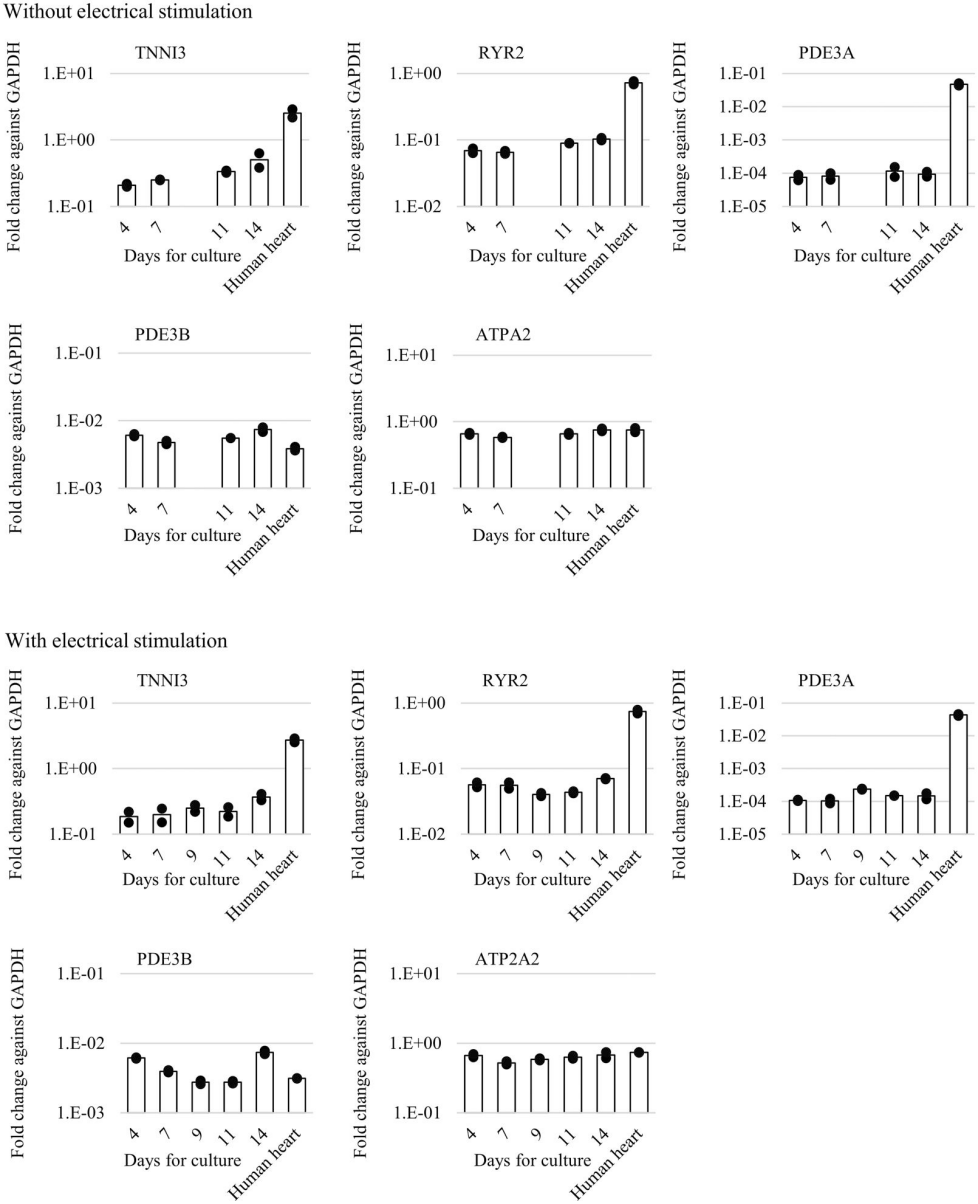
Gene expression in human induced pluripotent stem cell-derived cardiomyocytes (hiPSC-CMs) at different culture times, with or without the electrical stimulation from day 7 to 11, compared to human heart tissue. Due to practical constraints during the experiment, samples from day 9 without electrical stimulation were not obtained. Total RNA from four wells of cardiomyocytes was pooled for qPCR analysis, which was performed in duplicate. Gene expression levels were normalized to GAPDH expression. Bars represent the average gene expression, while open circles indicate individual qPCR sample values.

The expression levels of *TNNI3*, associated with sarcomere maturation, and *RYR2*, *PDE3A*, *PDE3B*, and *ATP2A2*, related to Ca^2+^ handling, were evaluated at various times during cell culture, both with and without electrical stimulation. We observed that neither the length of the culture nor the electrical stimulation had an impact on the selected gene expression levels (Fig. 8). The expression levels of *TNNI3* and *RYR2* increased slightly with extended culture time; however, they remained at approximately 1/10th of the levels observed in adult human heart tissue. The expression of *PDE3A* was approximately 1/1000th of the levels in the human heart. By contrast, the expression levels of *PDE3B* and *ATP2A2* were comparable to those found in the human heart.

### Morphological evaluation of hiPSC-CMs by transmission electron microscopy

**Fig. 9.**
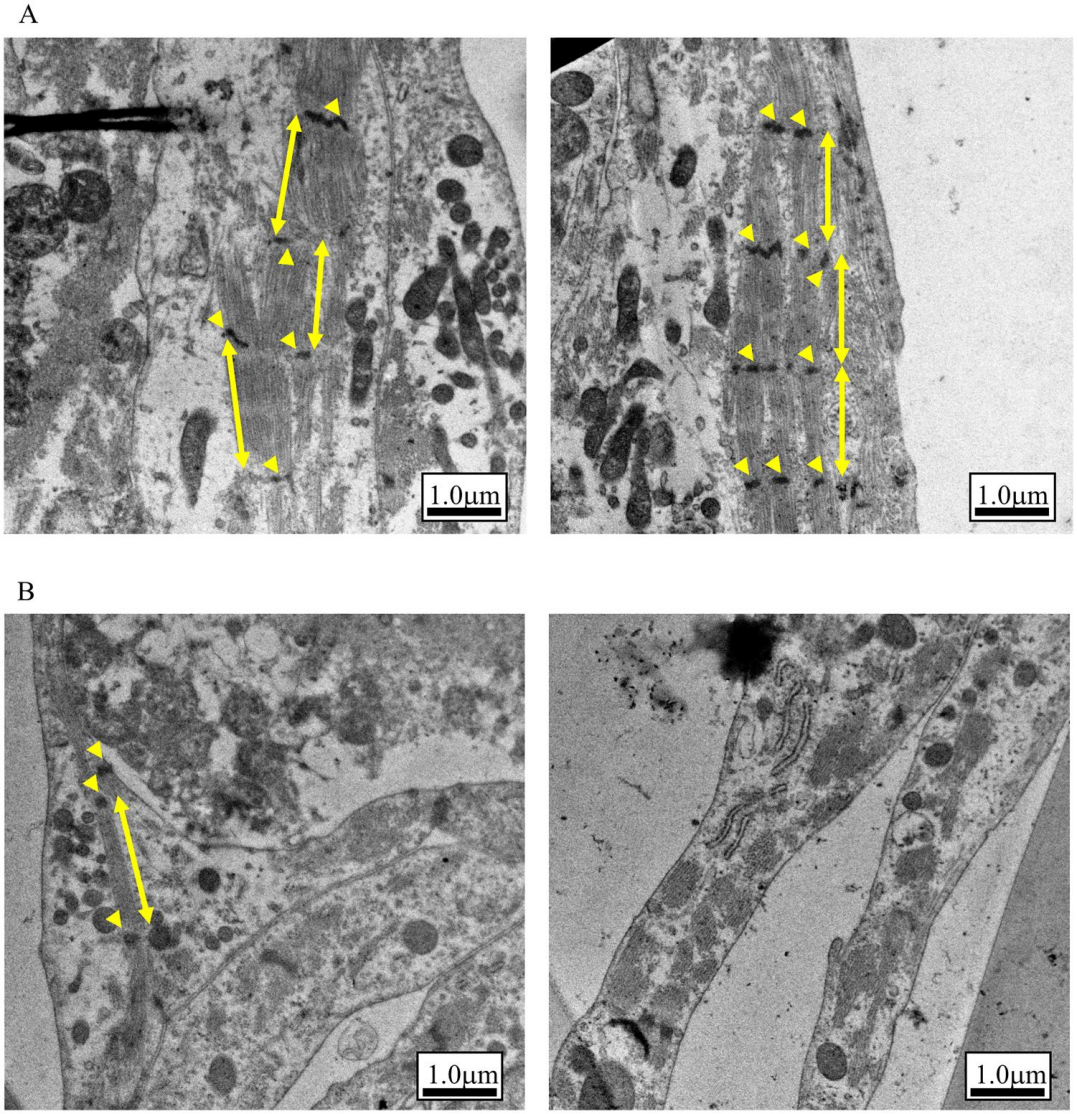
Visualization of intracellular organelles by transmission electron microscopy. A) Image of human induced pluripotent stem cell-derived cardiomyocytes (hiPSC-CMs) with electrical stimulation. B) Image of hiPSC-CMs without electrical stimulation. Arrowhead indicates Z-band. Two-way arrow indicates the area of sarcomere.

Sarcomere structures in the cultured hiPSC-CMs stimulated electrically were compared with those in unstimulated cardiomyocytes. Analyzing more than 40 images acquired at various magnifications (×5k, ×10k, and ×25k) for each condition, found that the electrically stimulated cells exhibited a more continuous orientation of sarcomere structures and more distinct Z-band than unstimulated cells, which displayed fragmented sarcomere structures and indistinct Z-bands. Representative images are shown in Fig. 9. These observations suggest that the electrical stimulation promoted the maturation of cytoskeletal structures in hiPSC-CMs.

### Assessment of the inotropic effect of compounds after electrical stimulation

The electrical stimulation enables the detection of positive inotropic effects caused by compounds such as isoproterenol (a nonselective β-adrenergic receptor agonist), forskolin (an adenylyl cyclase activator), (*S*)-(−)-BayK8644 (an L-type Ca^2+^ channel activator), and digoxin (a Na^+^/K^+^ ATPase inhibitor). By contrast, the stimulation did not alter the responses to the negative inotropic compound verapamil (an L-type Ca^2+^ channel blocker), as the effect can be detected even without electrical stimulation. It was also confirmed that the vehicle control (DMSO) does not affect cell contractility (Fig. 5 and Table 1).

### Assay performance for the inotropic effects of test compounds compared with vehicle control

We detected significant inotropic effects compared with the vehicle control for five known positive inotropic compounds: Isoproterenol, dobutamine, forskolin, (*S*)-(−)-Bay K8644, and digoxin. The other four positive inotropic compounds, including ouabain (a Na^+^/K^+^ ATPase inhibitor), milrinone (a PDE-3 inhibitor), pimobendan (a calcium sensitizer with PDE-3 inhibition), and omecamtiv mecarbil (a myosin activator), tended to increase the beat amplitude of cardiomyocytes; however, we found no significant differences compared with the vehicle control. Three compounds (isoproterenol, milrinone, and pimobendan) were tested at two different laboratories. The results obtained were similar in terms of increased beat amplitude of cardiomyocytes, but significant differences were observed at only one of the facilities. We also tested known negative inotropic compounds: Verapamil (an L-type Ca^2+^ channel blocker), mexiletine (a Na^+^ channel blocker), and propranolol (a nonselective β-receptor antagonist). Compared to the vehicle control, verapamil caused a significant concentration-dependent decrease in cardiomyocyte beat amplitude. In contrast, mexiletine and propranolol also reduced the beat amplitude, but these decreases were not significant (Fig. 6 and Table 2).

### Assessment of the potential to prolong QT intervals and induce proarrhythmia

We confirmed that E-4031, a well-known hERG potassium (Kv11.1) channel blocker, prolongs the field potential duration (FPD)—an indicator of the QT interval—in a dose-dependent manner and induces early afterdepolarization, a marker of proarrhythmia, at high concentrations. Conversely, verapamil exhibited a dose-dependent shortening of the FPD. These results demonstrate that hiPSC-CMs retain their ability to detect the potential for prolonging the QT interval and inducing arrhythmia after electrical stimulation (Fig. 7). Isoproterenol, a known positive inotropy, did not affect the corrected field potential duration (FPDc), which accounts for beat rate, in this study.

## Discussion

In the present study, we provide evidence that 48 h of electrical stimulation induces partial maturation of the functional and morphological properties of hiPSC-CMs. It is well established that human cardiomyocytes acquire a more mature phenotype through continuous electrical stimulation during development compared to conventional culture (Martherus et al. 2010). The force–frequency relationship (FFR) is widely recognized as an indicator of maturity because it reflects calcium handling and contractile mechanisms (Wiegerinck et al. 2009). Adult cardiomyocytes exhibit a positive FFR, characterized by an increase in contractile force with increasing stimulation frequency. Conversely, infant cardiomyocytes display a flat or negative FFR, indicating little to no change in contractile force as stimulation frequency increases (Wiegerinck et al. 2009). The present findings confirm that hiPSC-CMs undergo partially functional maturation with respect to FFR after the electrical stimulation, as evidenced by the transition from a negative to a partially positive FFR (Fig. 2). Transmission electron microscopy revealed that hiPSC-CMs subjected to the electrical stimulation showed slight improvements in sarcomere orientation and Z-band formation (Fig. 9). In contrast, the electrical stimulation did not induce any changes in intracellular Ca^2+^ concentration upon excitation (Fig. 4). Similarly, gene expression levels related to functional maturation, including those associated with sarcomere formation and Ca^2+^ handling, were unaffected by the treatment (Fig. 8).

The low expression level of PDE3A in hiPSC-CMs is consistent with previous reports (Okai et al. 2020; Satsuka et al. 2024). The acquisition of partially matured FFR is likely due not to changes in molecular expression profiles, but rather to functional or structural improvements, such as enhanced coupling of Ca^2+^ transients to contractile force or improved alignment of contraction direction. This conclusion is further supported by the transient nature of the partially positive FFR induced by the electrical stimulation: Although the effect persists for at least 2 h after stimulation ceases, the FFR returns to a completely negative state 5 d after the treatment ends (data not shown). Additionally, it should be noted that the limited understanding of the maturation status of hiPSC-CMs after the electrical stimulation represents a limitation of the current study. Future research employing RNA sequencing and other molecular analyses is necessary to evaluate the full spectrum of biological changes induced by the process.

Considering that electrical stimulation induces unidirectional propagation of the depolarization signal from the stimulation electrode through the cardiac cell sheet, it is reasonable to suggest that this process strengthens electrical signal conduction, which may explain the observed improvement in conduction velocity (Fig. 3). To exhibit a positive force–frequency relationship, the depolarization signal likely needs to propagate sufficiently quickly throughout the cardiac cell sheet to enable a coordinated response to rapid stimulation; otherwise, the effects of individual cellular contractions may cancel each other out. Therefore, one possible explanation for the acquisition of a positive force–frequency relationship following electrical stimulation is an improvement in conduction velocity, although this hypothesis remains tentative.

It has been reported that the peak force in response to stimulation frequency in the adult human heart occurs at around 3 Hz (Mulieri et al. 1992). However, in this study, the peak force was observed at 1.5 to 2.0 Hz, indicating that the functional maturation achieved here remains below that of adult cardiomyocytes. Similarly, sarcomeres within the cardiomyocytes are sparse, and their structures remain fragmented and much less organized compared to adult cardiomyocytes (Burbaum et al. 2021), even after the electrical stimulation. On the other hand, the improved conduction velocity observed in this study ranged from 0.4 to 0.5 m/s, which approaches the reported conduction velocity of human myocardium (0.3 to 0.4 m/s) (Raymond et al. 2009).

We compared the effects of the electrical stimulation on the force–frequency relationship (FFR) (Fig. 2) and conduction velocity (Fig. 3) at two independent facilities. Electrical stimulation induced a positive FFR up to frequencies of 1.5 to 2.0 Hz at both facilities. Similarly, an improvement in conduction velocity following the electrical stimulation was observed at both facilities. It was confirmed that the similar functional improvements elicited by the electrical stimulation are observed across different faciilties. However, differences in the cardiac functions after electrical stimulation were noted between the two facilities, as reflected by variations in the frequency at which peak beat amplitude occurred and baseline conduction velocity. These discrepancies are likely attributable to differences in hiPSC-derived cardiomyocyte product lots, culture media, or culture conditions such as precise cell density. Understanding how these factors influence the baseline state of cardiac cell sheets, the response to electrical stimulation, and subsequent drug responses will be essential for achieving more precise assay quality control.

We confirmed that detecting positive inotropic effects induced by pharmaceutical compounds via beat amplitude measurements using the MEA system becomes possible following the electrical stimulation. This was demonstrated with compounds targeting various mechanisms, including β-adrenergic receptor modulation, Na^+^/K^+^-ATPase inhibition, adenylyl cyclase activation, and L-type Ca^2+^ channel modulation. In contrast, negative inotropic effects—such as the concentration-dependent decrease in beat amplitude caused by the L-type Ca^2+^ channel inhibitor verapamil—were observed even without electrical stimulation (Fig. 5).

Based on these observations, we evaluated the system’s ability to detect the inotropic effects of pharmaceutical compounds at both facilities (Fig. 6 and Table 2). At facility A, significant inotropic effects were observed for various compounds compared to the vehicle control (DMSO). These compounds act through diverse mechanisms, including β-adrenergic receptor agonists (isoproterenol and dobutamine), an adenylyl cyclase activator (forskolin), an L-type Ca^2+^ channel activator ((S)-(-)-BayK8644), a Na^+^/K^+^ ATPase inhibitor (digoxin), and an L-type Ca^2+^ channel blocker (verapamil). Although PDE-3 inhibitors (milrinone and pimobendan), the myosin activator (omecamtiv mecarbil), and the Na^+^ channel blocker (mexiletine) did not produce statistically significant differences compared to the vehicle control, consistent trends in beat amplitude changes were observed across all three mechanisms, aligning with their expected pharmacological effects. At facility B, increases in beat amplitude were observed following exposure to the β-adrenergic receptor agonist isoproterenol, the Na^+^/K^+^ ATPase inhibitor ouabain, and the PDE-3 inhibitors milrinone and pimobendan, although these changes were not statistically significant compared to the vehicle control. These trends are consistent with the results observed at facility A and the known pharmacology of these compounds. However, for several compounds at both facilities, no statistically significant differences from the vehicle control were detected. Increasing the number of replicates in future experiments may provide more definitive conclusions regarding the assay’s ability to detect these inotropic effects. The inotropic effects and concentration ranges detected in this study are consistent with previously reported effects observed in hiPSC-CM systems (Scott et al. 2014; Okai et al. 2020; Zhang et al. 2023; Satsuka et al. 2024) and human cardiomyocytes (Abi-Gerges et al. 2020). For example, omecamtiv mecarbil increased beat amplitude up to 1 µM but decreased it at 3 µM, aligning with earlier reports of effective concentrations between 200 nM and 600 nM (Malik et al. 2011; Feric et al. 2019; Zhang et al. 2023). It should be noted that the concentration range at which isoproterenol exerts its effects varies depending on the detection method. For example, effects on cardiomyocyte contraction velocity can be detected at nanomolar concentrations, whereas cellular deformation is detectable at higher concentrations ranging from 10 to 100 nM (Satsuka et al. 2024). The higher concentration range at which we observed isoproterenol-induced changes in beat amplitude (approximately 300 nM) is likely due to the specific detection method used in this study.

We confirmed that electrophysiological measurements can also be performed following the electrical stimulation. The observed responses to the hERG inhibitor E-4031 and the L-type Ca^2+^ channel blocker verapamil aligned with previous reports (Ando et al. 2017; Blinova et al. 2018). Additionally, the isoproterenol-induced decrease in corrected field potential duration (FPDc) is consistent with earlier findings demonstrating a reduction in QTc using the Langendorff assay (Isobe et al. 2018). Although we did not observe a clear improvement in the electrophysiological assessment of drug effects specifically attributable to the electrical stimulation, this approach remains valuable because it enables simultaneous evaluation of both contractility and electrophysiology. Combining these measurements within a single system offers more comprehensive insights into the integrated effects of compounds on cardiomyocyte electrophysiology and contractility, while also reducing material usage and resource consumption—advantages that are particularly important for early-stage drug discovery.

In summary, this assay reveals various pharmacological effects on cardiac contractility, though some mechanisms are less sensitive. Unlike other hiPSC-CM assays, it provides electrophysiological insights on the same platform. Given the challenges in consistent drug exposure across experiments, this integrated approach offers clear advantages by assessing both contractility and electrophysiology simultaneously. While animal experiments and assays using animal or human tissues provide tissue- or organ-level insights, this assay supports early-stage drug discovery by requiring less effort, minimal compound amounts, and lower costs.

This study showed that 48 h of electrical stimulation in the MEA system partially enhances hiPSC-CM functional and morphological maturation. Additionally, it enables evaluation of inotropic effects from diverse compounds, allowing concurrent assessment of both inotropic and electrophysiological drug effects, making it a valuable early drug discovery tool.
